# Recent Advancements in Receptor Layer Engineering for Applications in SPR-Based Immunodiagnostics

**DOI:** 10.3390/s21113781

**Published:** 2021-05-29

**Authors:** Marcin Drozd, Sylwia Karoń, Elżbieta Malinowska

**Affiliations:** 1 Faculty of Chemistry, The Chair of Medical Biotechnology, Warsaw University of Technology, Noakowskiego 3, 00-664 Warsaw, Poland; sylwia.karon.dokt@pw.edu.pl; 2 Center for Advanced Materials and Technologies, Warsaw University of Technology, Poleczki 19, 02-822 Warsaw, Poland

**Keywords:** SPR, immunosensor, antibody immobilization, medical diagnostics, gold functionalization, non-specific interactions, self-assembled monolayers

## Abstract

The rapid progress in the development of surface plasmon resonance-based immunosensing platforms offers wide application possibilities in medical diagnostics as a label-free alternative to enzyme immunoassays. The early diagnosis of diseases or metabolic changes through the detection of biomarkers in body fluids requires methods characterized by a very good sensitivity and selectivity. In the case of the SPR technique, as well as other surface-sensitive detection strategies, the quality of the transducer-immunoreceptor interphase is crucial for maintaining the analytical reliability of an assay. In this work, an overview of general approaches to the design of functional SPR-immunoassays is presented. It covers both immunosensors, the design of which utilizes well-known and often commercially available substrates, as well as the latest solutions developed *in-house*. Various approaches employing chemical and passive binding, affinity-based antibody immobilization, and the introduction of nanomaterial-based surfaces are discussed. The essence of their influence on the improvement of the main analytical parameters of a given immunosensor is explained. Particular attention is paid to solutions compatible with the latest trends in the development of label-free immunosensors, such as platforms dedicated to real-time monitoring in a *quasi*-continuous mode, the use of in situ-generated receptor layers (elimination of the regeneration step), and biosensors using recombinant and labelled protein receptors.

## 1. Introduction

### 1.1. Advantages of SPR-Based Immunosensing

SPR detection is classified as a label-free approach in which the generation of a measurable signal is directly related to the act of binding the analyte by the receptor layer. This attribute bestows the universality of SPR biosensors, since each molecule bound to the surface, regardless of its chemical character, can be considered as a specific signal carrier. Thus, direct detection of interactions by monitoring changes in the surface plasmon resonance seems to be perfectly tailored to the signal transduction of affinity sensors, with immunosensors at the forefront. This is an undoubted advantage over the methods typically used in medical diagnostics, like immunoassays labelled with enzymes, fluorophores, radioisotopes, and nanomaterials, since in these methods, the introduction of an additional labelling is inevitable. The need for the conjugation and purification of secondary bioreceptor complicates and extends the whole analysis. In turn, the extra steps of antibody/conjugate binding and enzymatic or catalytic reaction are often required [1]. What is more, for the most common sandwich assay format, the use of a previously selected and matched antibody pair is required, which is not always achievable for small or unusual antigens. In such cases, the advantages of SPR-based approaches are the most visible. Label-free methods are easier to design, thanks to the simplicity of a sensing mechanism that naturally mimics the occurring interactions of antibodies with specific antigens. In turn, the affinity-based mass association on the sensor surface, which induces the change in the local refractive index is convenient for optical signal acquisition by tracking the associated SPR resonant angle in a label-free and real time format. Thereby, the analysis of interactions with layers of immobilized antibodies or antigens on SPR transducers also provides auxiliary information about the thermodynamic aspects of the immunoreaction and binding kinetics. Thanks to this, over 30 years from its first application (1990), SPR has become the gold standard for studying biological interactions and—apart from being used for direct signal transduction—also acts as a reference tool, which is indispensable in the development of other classes of affinity biosensors [2].

Molecules within the range of the SPR evanescent wave generated at the interface between metal (typically gold) and the dielectric (typically a liquid medium—buffer or tested sample) are responsible for the change of the refractive index (RI), and thus, the SPR signal depends, to a certain extent, on several factors [3]. The main factor is related to the molecular mass of the bound object, which is mirrored in changes of the local RI. The distance of the captured molecule from the Au surface is also important. The effect of analyte binding on the change in the SPR resonant angle is the most prominent in the immediate vicinity of the metal, and it decays exponentially as it moves away from the plasmonic metal surface. For this reason, an analytically useful range of SPR is not more than 200 nanometers for the classic SPR wave generated on macroscopic, flat transducers [4] and several tens of nm (~30 nm) for the localized SPR occurring at the surface of nanoparticles [5].

Modern medical diagnostics, ranging from screening tests for the rapid detection of civilization diseases’ biomarkers to the detection of viral and bacterial infections, is largely based on the detection of specific, rare proteins [6,7,8,9,10]. A common feature of most of these types of molecular targets is their relatively large mass and wide availability of high-affinity antibodies for their detection. Both features make protein biomarkers attractive SPR targets, providing the possibility for their direct determination. In a classic format, antibodies play the role of ligands, and an analytical signal results directly from the act of the specific binding of the target antigen. This is the preferred format, as it enables detection directly in samples without additional steps, either during the sample preparation or during the analysis. However, this does not change the fact that in many cases, it is possible to use indirect formats, such as a sandwich or competitive/inhibition SPR assay. In most cases, it is dictated by the low molecular weight of the analyte or its very low concentration, resulting in an insufficient sensitivity of the direct assay [11]. However, the immobilization of an antigen is not always forced by circumstances, and it can be chosen, e.g., when this format allows for its convenient attachment, which is often the case with labelled recombinant proteins. Due to the high availability of universal coupling agents and protein conjugation methods, it is possible to easily adapt the format of the SPR assay, depending on the requirements of the specific analysis.

SPR detectors, like most of the label-free ones available today, do not distinguish specifically bound analytes from randomly adsorbed interferents. For this reason, SPR biosensors are particularly prone to problems with non-specific binding during analyses of complex samples and the determination of the ultra-low concentration level of the analytes, which are common for medical immunodiagnostics. Problems with the adsorption of body fluid sample components may be reflected in the deterioration of key performance parameters, such as baseline stability, limit of detection (LOD), and lifetime [12]. An insufficient fouling resistance of the immunosensing layer disturbs the sensitivity and selectivity of the assay defined in biomedical terms, which is understood as the percentage of false positive and false negative results and may result in a failure of their validation. This is why so much attention is paid to the appropriate quality of SPR intermediate immunosurfaces for application in medical diagnostics in the literature [13].

**Figure 1:**
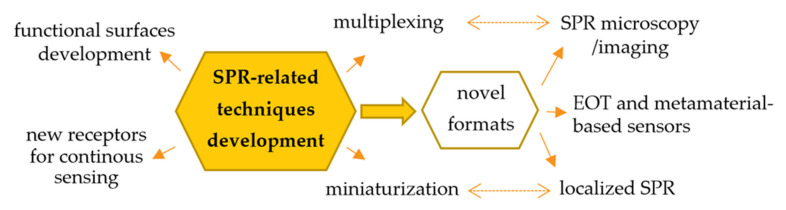
Current major trends in the development of plasmon-based immunosensors.

Since its first application, a number of methods have been developed to employ SPR for the sensitive detection of bioanalytes in complex samples. The current and main trends in the development of the SPR-related techniques for biosensing are focused on:

- the development of universal substrates for immunosensing, enabling the simple and cheap fabrication of high-quality receptor layers, aimed at improving the sensitivity and selectivity of sensors [14];
- novel approaches to signal amplification, which are particularly important in the case of the determination of bioanalytes occurring at ultra-low concentration levels and low molecular weight targets [15];
- new strategies of plasmonic signal transduction (single-particle and transmission LSPR sensors, Extraordinary Optical Transmission (EOT)-based sensors utilizing plasmonic nanopores, or sensors using meta-surfaces and metamaterials) [16,17,18];
- possibility for the simultaneous determination of more than one analyte (multiplexing);
- miniaturization of detectors, development of portable SPR systems for the *on-site* detection and adaptation of commonly available instruments to the role of detectors (e.g., integration of SPR platforms with smartphones) [19];

The ongoing development of SPR-based biosensors, both in the context of improving key parameters of receptor layers, as well as methods and instruments for data acquisition, expands the application possibilities of SPR and related techniques in medical analysis. These aspects are comprehensively discussed in recent reviews [1,5,15] and graphically summarized in Figure 1.

### 1.2. Antibodies as Affinity Bioreceptors—History and Recent Trends

Immunological receptors are commonly believed to be the most powerful recognition elements for biosensing. Due to their unsurpassed affinity and specificity of interaction with a variety of molecular targets and universality, antibodies have become the flagship biological receptors, with by far the broadest application in clinical analysis and biosensors construction [20]. In 1890, von Behring and Kitasato reported the existence of an agent in the blood capable of neutralizing diphtheria toxins, and they called it “Antikörper”, which was later translated as “antibody.” Antisomatogen is the name given to the material that causes the formation of antibodies, and the word “antigen” is a contraction of this expression. More than a century of research into the structure and function of antibodies has highlighted their complexities. Antibodies possess many unique properties, such as a high diversity (binding to a wide range of targets), specificity, and structural integrity. While individual immunoglobulins bind a narrow and limited set of ligands, as a population, they are capable of binding to an almost infinite number of antigens of little or no similarity [21]. Immunosensors are based on the concept of forming complexes, which are a product of the interaction between antibodies and their targets. Antibodies were first used in sensing applications in the late 1950s. Their rapid kinetics, specificity, and ability of antigen-binding in both artificial media and real samples caused their extreme popularity as major parts of receptor layers [22]. Antibody–antigen binding is mediated by the existence of amino acids at the paratope–epitope interface. It has long been debated whether the interaction between the paratope of the immunoglobulin and antigen epitope is predictable, as this breakthrough would completely change medical biotechnology. Nevertheless, this is currently unknown [23]. The most frequently utilized antibody isotype is immunoglobulin G (IgG), both mono- and polyclonal. The first one, which is able to bind only a single epitope, had been the most expensive and desired for years. Nowadays, it has changed due to the development of genetic engineering techniques and worldwide concern for animal rights, which resulted in them becoming cheaper. By contrast, polyclonal antibodies are becoming rarer. Moreover, to avoid batch-to-batch variations, which are common for antibodies produced in animals, antigen-binding fragments (Fab) produced in bacteria are used, instead of whole immunoglobulins [24,25]. The expansion of alternatives to classical antibodies led to the development of novel techniques for their manufacturing, isolation, and selection, such as phage display [26], artificial cell surface constructs and production in plants [27], generation of single-chain fragment variable (scFv) [28], among others [29]. Moreover, new types of protein binders have emerged, such as affimers, which are able to withstand a wide range of temperatures and pH levels [30], or camelid nanobodies—the smallest naturally-derived fragments, which can bind an antigen [31]. While there are more replacements for classical or recombinant antibodies and their fragments, such as aptamers [32], common immunoglobulins are still on top. Their main advantages are their availability, cheapness, and facility of their conjugation and tagging.

The number of articles that are published on the topic of SPR immunosensors has rapidly grown in the last few years. The dataset retrieved from the Web of Science database on 22 May 2021 was based on the following search terms: (*SPR* OR *surface plasmon resonance* AND *immunosensor* OR *immunosensing*). It includes 7803 references over the last 5 years (2017–2021), of which most are in the last 2 years (nearly 2000 each). The advancements in the field of nanomaterial-based LSPR immunosensors, new formats, and designs of receptor layers, as well as methods of signal enhancement for sensitive plasmon-based sensing, are behind this intense increase in scientific output.

### 1.3. Main Challenges for SPR Immunosensors in Biomedical Diagnostics

Biosensing in body fluids, such as serum or blood plasma, is challenging due to the occurrence of an undesirable non-specific adsorption, resulting from the complexity of the sample matrix [12,33]. To provide clinically relevant results, numerous strategies to improve the analytical parameters of SPR sensors are currently being developed, among which two main trends can be distinguished. The first utilizes the amplification of the affinity-based signal by introducing additional labelling steps (e.g., with secondary antibodies, bioconjugates of plasmonic nanoparticles, and other particles capable of generating changes in the local refractive index). This strategy has very good results in the case of low-molecular mass analytes, but it takes place at the cost of an increased complexity of the measurement procedure and the label-free detection format. The second approach is focused on the broadly understood improvement of the properties of substrates and layers that play the role of a transducer-receptor interphase. A number of examples have been described in the literature, in which the appropriate architecture of the receptor layer’s substrate affects various aspects of the SPR sensors, starting from the improvement of the sensitivity of detection (increase of SPR angle shift), through extending the surface available for bioreceptor binding, and ending with lowering background signals by developing layers with an ultra-low nonspecific adsorption [12,34,35]. The surface-related approach seems to be a challenge of particular importance from the point of view of SPR immunosensors, as it allows the most convenient, label-free working principle to be maintained. The development of high-quality immunosensing platforms allows us to fully reveal their greatest potential, i.e., their application for rapid and real-time diagnostic purposes.

The selection of an adequate immobilization strategy is vital for the success of the whole SPR biosensing platform. The receptor layer for applications in the analysis of complex samples, which meets the requirements of modern medical diagnostics, must be a compromise between various aspects affecting its analytical and working parameters. The most important issues cover:

(I) The metal-receptor interphase thickness—the introduction of linkers/spacers ensuring the separation of the immunoreceptor from the surface has a positive effect on the thermodynamics and kinetics of the antibody–antigen interaction. It also facilitates the surface passivation and minimization of the impact of non-specific binding. However, an excessive distance of the biosensitive layer from SPR-active surface results in a significant decrease in sensitivity.
(II) The surface density and architecture of bioreceptors—a high receptor density represented by protein binding capacity of the interphase is always desirable due to the increased sensitivity of the immunosensor. However, the use of very dense receptor layers with an extensive 3D architecture may generate steric hindrances and problems with mass transport, affecting the response time and the ability of sensor regeneration.
(III) The method of the immobilization of recognition elements—compounds used to passivate the transducer surface should also offer the possibility of a robust, covalent attachment of the Ab receptor layer. On the other hand, site-oriented immobilization via affinity interactions (the use of recombinant/tagged receptors or auxiliary proteins, such as superantigen-like proteins A, G, and A/G) supports the preservation of the native form of receptors and the appropriate orientation and exposure of binding sites.

Both the classic SPR on the surface of macroscopic transducers, multiplexed SPR imaging (SPRi) assays, and the newest plasmonic technique—localized SPR on the surface of nanomaterials—have become attractive platforms for the construction of immunosensors for medical diagnostics [17,36]. The mentioned methodologies of SPR-based immunosensing, regardless of the receptor layer design, offer specific advantages and have different limitations in terms of detection performance. Classic SPR sensors, thanks to the possibility of differential analysis with the use of a reference cell, enable the efficient suppression of various types of interferences, both instrumental and related to surface processes. In addition, such devices typically work in the angular scan mode, which offers the best sensitivity to detect shifts in the resonance angle in a wide range. However, it can be achieved at the expense of the complexity of the detection system. SPRi immunosensors provide attractive platforms for the simultaneous detection of multiple analytes, with the spatially resolved detection of reflectivity changes. It is typical for this type of device detection systems, which are based on the analysis of images captured by a CCD camera, to offer a slightly lower detection sensitivity than an angular scan. In turn, the main advantage of immunosensors based on the localized plasmon resonance of nanostructures (LSPR) is the simplicity of the detection system based on transmission measurements by recording the absorbance spectrum or even by tracking changes in the absorbance intensity at a fixed wavelength. The analysis of the shift of the LSPR maxima requires a high resolution of detectors, but they are much easier to miniaturize. Unfortunately, due to the short range of the LSPR phenomenon, the possibilities of designing immunosurfaces with a high anti-fouling resistance are limited. However, thanks to the appropriate modification and use of signal amplification, it is possible to detect small and low-abundant analytes by means of LSPR immunosensors [5,18].

Biosensors exploiting the unique plasmonic properties of gold, regardless of the employed format and the signal generation and detection mechanism, require a careful design of the receptor layer. Therefore, apart from the intensively developed new detection formats, such as the use of gold nanoparticles as plasmonic transducers in LSPR biosensing, the design of new functional immunosurfaces is still one of the main scientific challenges. It is worth noting that in recent years many types of label-free immunosensing platforms founded on Au, Ag, or metal oxides have been developed. These cover, e.g., piezoelectric biosensors based on quartz crystal microbalance, and ultra-sensitive optical sensors employing surface-enhanced Raman scattering. The SERS phenomenon, thanks to the strong amplification of the signal of specific molecules on the sensor substrate by plasmonic effects, enables the construction of sensors for the detection of bioanalytes characterized by very low detection limits [7,8].

In a further part of this review, the current state of the art of gold surface modifications for SPR and other plasmonic-based immunosensing platforms is discussed. The review focuses on the solutions developed in recent years and the most popular previously developed approaches in the field of SPR intermediate layers for antibodies and antigens immobilization—ranging from commercial solutions, through novelties in the field of self-assembled monolayers, the latest achievements in the field of chemical and affinity-based immobilization, and nanomaterial-based layers for SPR signal amplification, to smart layers for multiplex immunosensing. The latest developments and trends in the field of universal platforms for semi-continuous plasmonic biosensors will be also discussed. Special attention will be paid to design solutions for and applications of SPR immunosensors in a direct and competitive format for the direct detection of bioanalytes of high biomedical importance, such as protein biomarkers, metabolites, or toxins in complex samples, such as body fluids.

## 2. Functional Surfaces and Non-Oriented Ab Immobilization Strategies for SPR Immunosensing

A consistent classification of the currently available methodologies for the design of functional SPR immunosurfaces is not an easy task, because the engineering of such interphases is a multistep process. Beyond the type of molecules and other components, there are numerous methodologies for attaching them to the surface of bare gold chips. Additionally, a number of protein receptor attachment strategies are offered, including non-oriented immobilization via adsorption, non-oriented immobilization via chemical coupling, and affinity-based, oriented immobilization of native and labelled receptors. The type of intermediate layer used was chosen as the basic classification criterion within this work. However, in the case of solutions that are difficult to classify (e.g., hybrid approaches, such as a combination of monolayers and affinity-based immobilization), the review goes beyond the adopted framework, and the described solutions are discussed in a broader context. In order to compare the most significant and recent research developments in this field, methods based on physical/chemical interactions and affinity-based approaches have been compiled in separate tables that summarize the individual groups of subchapters.

### 2.1. Passive and Non-Covalent Adsorption

Methods based on the adsorption of antibodies and antigenic receptors on surfaces are widely used in the construction of immunoassays due to their simplicity of implementation. Even a bare gold surface shows the ability to bind immunoglobulins and their fragments [37,38]. A much wider range of possibilities for protein adsorption is offered by transducers pre-modified with cationic SAMs and polymer layers, e.g., cysteamine, APTES, and PDDA [39,40]. The binding of receptors to the surface is mainly determined by electrostatic interactions between the oppositely charged terminal ammonium and carboxylate residues of aminoacids, as well as coordination-type interactions. In another approach, a mechanism based on the interaction of hydrophobic protein domains allowed for the immobilization of antibodies on SPR substrates coated with hydrophobic polymers. Thin polystyrene layers were used in the construction of SPR immunosensors to bind antibodies and selected types of antigens (e.g., virus particles) by Wan der Waals hydrophobic forces [41]. However, due to the high risk of the inactivation of sensitive protein recognition elements, as a result of conformational changes during adsorption on the surface, as well as difficulties in the fabrication of repeatable and homogeneous layers with a high resistance to non-specific adsorption, such approaches are losing importance in SPR-based applications. Towards the development of durable and resistant immunolayers dedicated to applications in flow conditions commonly used in SPR, methods using random immobilization via chemical coupling and oriented affinity-based immobilization are currently dominating [42].

### 2.2. Covalent Anchoring and Surface Passivation through Self-Assembled Monolayers (SAMs)

While antibodies themselves have the ability to spontaneously adsorb on the Au surface and chemisorb through terminal cysteine residues (previously converted to the more chemically active reduced form or after the previous derivatization of terminal lysine residues by means of Traut’s reagent [38,43]), these methods are quite rarely used in the construction of SPR sensors. The main disadvantage of such an approach is the deterioration of the Ab binding capacity due to conformational changes caused by the multidentate interaction with the surface of gold. What is more, such layers are characterized by a poor homogeneity, moderate reproducibility, and tendency to desorb under shear stress in microfluidic conditions. Therefore, intermediate layers that allow for covalent coupling with functional groups of a protein receptor are commonly used [44]. Primary amine residues, which are more abundant and less demanding in terms of the coupling reaction conditions than thiol/disulfide moieties, are most often employed for this purpose.

The easiest approach to the formation of functional SPR interphases reactive in amine-coupling reactions is the use of the self-assembly of bifunctional, -COOH terminated thiols [45]. Due to the better passivating properties and ability to form highly ordered molecular assemblies without pinholes and defects, the most commonly used are linkers with a long aliphatic chain—mercaptoundecanoic acid (MUA) or mercaptohexadecanoic acid (MHDA) [46,47,48,49,50]. Detailed considerations on the impact of the used *n*-alkanethiolate chain on the capacity of antibody immobilization and antigen-binding efficiency can be found in the research provided by Bhadra et al. [51].

**Figure 2:**
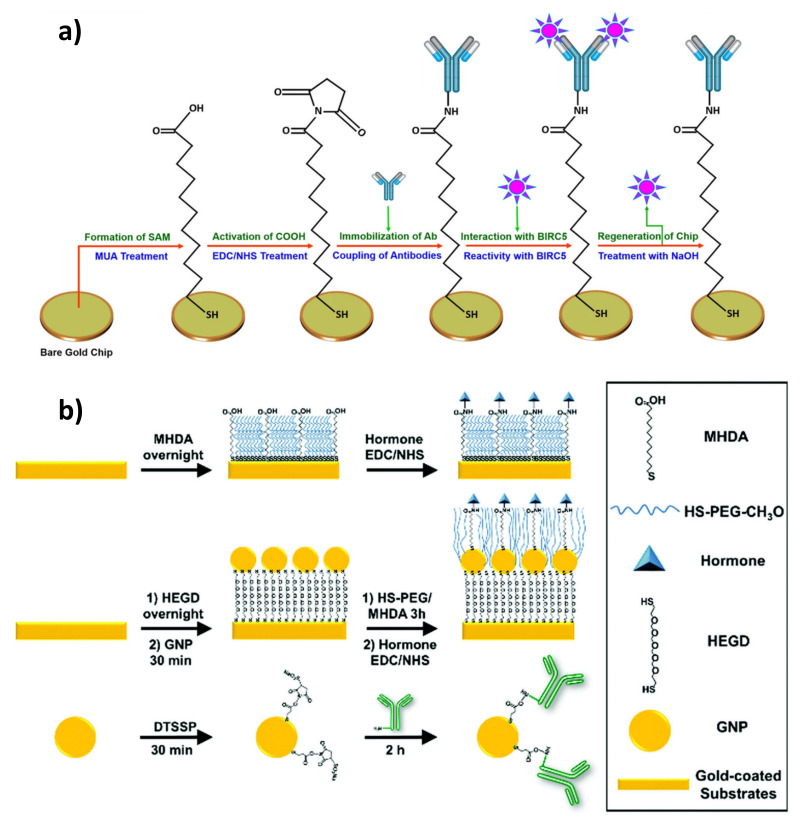
Examples of the utilization of various *n*-alkanethiolate and mixed SAMs in SPR immunosensing. (**a**) Schematic representation of ω-carboxylic acid-based SPR immunosensor fabrication with the use of EDC/NHS coupling, (**b**) various bifunctional linkers used for gold surface functionalization: MHDA/mercaptoundecanol mixed SAM (top), HEGD SAM for AuNPs self-assembly (middle), and DTSSP linker-mediated conjugation to AuNPs. Adapted with permission from Jena et al. [49] (under the terms and conditions of the Creative Commons CC BY License) and Castiello et al. [53] (Copyright © (2019) Royal Society of Chemistry).

Beyond ω-mercaptocarboxylic acids, in the design of SPR immunosensors, thiol-terminated amine-reactive prolinkers with terminal NHS ester groups, such as Cr5B and DSP (disuccinimido dithiobispropionate), are also exploited. The formation of such SAMs can be followed by a single-step immobilization of antibodies, without the need for prior activation [52,53]. Apart from bifunctional molecules, simple *n*-alkanethiols are also used in the construction of SPR sensors. Layers based on *n*-octanethiol were employed to passivate a gold surface or immobilize low-molecular, non-polar inhibitors, and antigens of non-protein nature by hydrophobic interactions [54,55]. Various strategies exploiting thiol-based SAMs in the construction of immunosensors for the detection of clinically relevant analytes are presented in Figure 2.

Even better passivating and protecting properties against non-specific binding to the sensor surface are demonstrated by ligands with an internal, polar spacer, such as linear PEG6 [56], dithiolalkanearomatic PEG (S_2_PEGCOOH) [57], and thiolated PEG linker DSPEG2 [58]. Thanks to their complex structure, containing both linear aliphatic segments and flexible or bulky segments, such as a PEG/aromatic ring, such compounds combine the ability of alkanethiols to form highly oriented, dense assemblies, with flexibility and hydrophilicity and thus an anti-fouling capacity of ethylene glycol oligomers. This promotes the immobilization of antibodies by reducing the negative influence of steric hindrance [59]. Another way to optimally adjust the surface density of anchoring groups for the efficient immobilization of antibodies is the use of mixed monolayers, in which ω-mercaptoalcohols play the role of a diluent and a surface backfilling. Their presence reduces the surface charge density, thus facilitating its formation without the negative impact on the performance of the Ab coupling reaction, even for -OH/-COOH ratios significantly greater than 1:1, reaching even 1:10. Typically used compounds include mercaptoalcohols with an aliphatic chain length equal or lower than the length of mercaptocarboxylic linkers [60] or mixed layers of functional PEGs, like HS-PEG-OH/-COOH [57,61,62]. This approach facilitates the access of high-molecular-mass receptors (such as antibodies) to the surface. Terminal hydroxyl groups are responsible for the formation of hydration layers. Hydrogen bonds that form in contact with the aqueous samples effectively counteract the adsorption of matrix proteins. Backfilling agents of a longer chain than carboxylate anchors are rarely used and are mainly used for the immobilization of small receptors (immunosensors in a competitive format) and in biosensors requiring a very effective suppression of surface fouling. It should be stressed that long and flexible PEG chains, apart from the natural hydrophilic barrier, also create a steric barrier, which, additionally, hinders the organization of layers on the surface due to entropy effects [12]. A detailed discussion of the influence of SAM composition on the availability of anchoring groups on the surface and the relation between the design of the SAM surface chemistry and sensitivity of the competitive immunoassay for the detection of small analytes can be found in the work authored by Castiello et al. [56].

The same group developed two competitive SPRi immunosensor configurations for the detection of insulin, glucagon, and somatostatin. In the first approach, antigen was immobilized on a mixed thiolate SAM. To obtain a high passivation and thus antifouling properties, 16-mercaptohexadecanoic acid (MHDA) molecules were used as anchors, and thiol-terminated PEG (CH_3_O-PEG-SH) was introduced as a surface backfiller. The second format covers an additional introduction of the auxiliary layer of gold nanoparticles anchored by a hexa(ethylene glycol) dithiol (HEGD) monolayer on gold to enhance the sensitivity by plasmonic coupling (Figure 2b). Both approaches were aimed at the formation of surfaces compatible with the EDC/NHS immobilization chemistry and resistant to non-specific adsorption, thus enabling an ultra-sensitive, indirect detection of pancreatic hormones [53,56].

**Figure 3:**
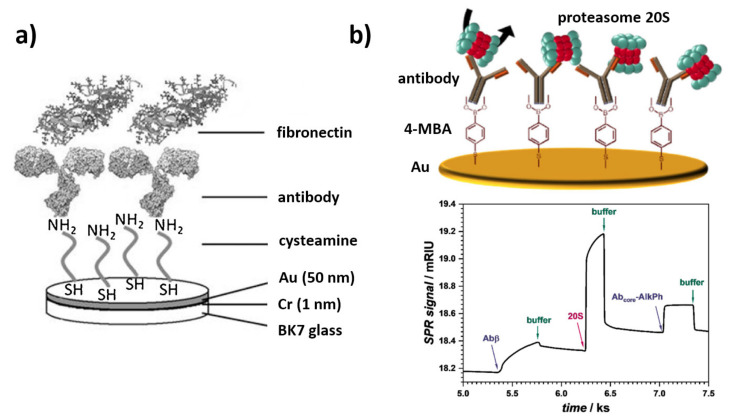
(**a**) The architecture of the oligopeptide SAM-based SPR immunosensor, (**b**) the mechanism of antibody immobilization through the interaction of carbohydrate residue with boronic acid SAM. Adapted with permission from Sankiewicz et al. [70] (Copyright © (2018) Elsevier) and Barsan et al. [71] (under the terms and conditions of the Creative Commons CC BY License).

Another method of coupling antibodies/antigens to the SPR chip surface is the use of SAMs with terminal amino groups. In this approach, 2-aminoethanethiol (cysteamine) is by far the most commonly used building block (Figure 3a) [63,64,65]. Despite the inability to form well-defined layers due to the insufficient length of the aliphatic chain, this ligand finds an application in the construction of immunosensors characterized by a validated reliability in typical medical samples. Terminal amino groups on the transducer surface open up a number of possibilities for the adsorptive and covalent attachment of protein receptors. The most important coupling agents compatible with -NH_2_-terminated interphases include glutaraldehyde (coupling with lysine residues through imine bond formation), EDC/NHS (coupling with carboxyl residues on C-terminal and the acidic amino acids of the protein), and maleimide (coupling with the thiol residues of cysteine in a reduced form) [66]. The limited resistance of amino-terminated SAMs towards protein adsorption is often associated with the necessity of using additional treatments, such as blocking of the immunosurface with polymers and proteins or the addition of anti-fouling agents (like dextrans and albumins) to the matrix buffers [46,47]. However, higher aliphatic aminothiols and functional aminothiols with oligoethylene glycol segments are not commonly used [67]. The smaller variety of the components used in the amino-SAMs can be explained by the significantly lower availability and high price of the higher aliphatic aminothiols, compared to their carboxyl counterparts.

Except for the simple, bifunctional thiol-based SAMs, bioinspired monolayers were also utilized in several examples of SPR interphases. An interesting approach is the use of monolayers of a short bifunctional peptide, 3-mercaptopropionic acid-His-His-His-Asp-Asp-COOH (3-MPA-HHHDD-OH). Such an oligopeptide has been introduced to reduce nonspecific adsorption in SPR-based PSA immunosensors, as described by Breault-Turcot et al. [68]. In a different approach, for the construction of universal biosensor platforms with a high resistance to non-specific protein binding, the synthetic layer of the carboxymethyl-terminated and disulfide-cored peptide was elaborated [69]. Short oligopeptides can be employed not only as a surface passivating agent. Islam et al. described the application of hexameric peptide ligands showing an affinity to human IgG as a foundation for an SPR immunosensor layer assembly. The authors demonstrated the Ab-binding capacity of the devised anchor to be similar to the well-known antibody-binding compound, protein A [67].

To minimize the risk of the abovementioned inactivation of antibodies due to the attachment via amine groups (abundant in the Fab region of immunoglobulins), other antibody functional groups were also harnessed as surface anchors. A very intuitive method described in the literature is based on the employment of EDC/NHS chemistry in an inverted format. Gorodkiewicz’s group used cysteamine SAMs for the construction of SPRi immunosensors. Carboxylic groups of antibodies in solution were first subjected to chemical activation, leading to the formation of reactive N-hydroxysuccinimide esters. Then, the pre-activated receptors react spontaneously with the surface amino groups [70,72]. Another method for the partially oriented immobilization of immunoglobulin G is the interaction of carbohydrates with phenylboronic acids (Figure 3b). IgG antibodies, as glycoproteins, contain N-glycan residues, located mainly in the Fc region, away from the binding sites. Such a configuration allows the appropriate spatial orientation of the immunoreceptors on the surface to be maintained [73]. SAMs composed of 4-mercaptophenylboronic acid (4-MBA) formed directly on gold [71] and 3-aminophenylboronic acid, immobilized on modified SPR transducers using graphene oxide carboxyl groups, have been described as compounds directly responsible for the capture of antibodies [74]. Additionally, amine-terminated ligands can be employed for carbohydrate-mediated immobilization through the previous oxidation of polysaccharide residues to aldehydes, followed by Schiff base formation [75].

### 2.3. Polymer-Based SPR Immunosensing Surfaces

Besides the abovementioned heterobifunctional thiols and short peptides, other compounds are also widely used as robust backfilling agents. Zwitterionic agents—both low-molecular-weight and polymeric—benefit from their well-defined charge distribution, ensuring a high hydrophilicity and suppressive properties against electrostatic interactions with proteins. Polymers such as betain and phosphorylcholine effectively fulfill their protective function, ensuring a very low, even undetectable level of protein adsorption (<10 ng/mm^2^) from samples such as serum or blood plasma [76]. However, their implementation in functional platforms for antibody immobilization is difficult, as it requires the preparation of mixed-type layers by thiolated anchor grafting [77]. Another approach is to use copolymers with zwitterionic segments, e.g., carboxybetaine acrylamide [78] and thiolated hyaluronic acid (HA) grafted with the zwitterionic CPPPPEKEKEKEK peptide [79]. Riedel et al. described the use of a coating with poly[(N-(2-hydroxypropyl)methacrylamide)-co-(carboxybetaine methacrylamide)] in the construction of a SPR sensor to detect hepatitis B antibodies in saliva. Previously grafted ω-mercaptoundecyl residuals were used as anchors to the chip surface and the antigen was attached using carbodiimide chemistry to the carboxylate terminal groups of the copolymer [78].

A number of hydrophilic polymers with functional groups capable of binding to a gold surface or groups that enable the chemical coupling of immunological receptors have been employed as interphases in SPR biosensors for clinically relevant analytes, including grafted hyaluronic acids [79,80], poly(N-isopropylacrylamide) (PNIPAAM) [63], poly(2-hydroxyethyl methacrylate) (pHEMA) [81], polysaccharides, and poly(β-peptoid)s. Hyperbranched polymers and dendrimers were also used as multifunctional immobilization platforms characterized by excellent anti-fouling properties. In work by Becherer et al., carboxymethylated dendritic polyglycidol grafted with thioctic amine was employed in the design of a sensor for the detection of anti-amyloid beta (Aβ) 1–40 antibodies [82]. In turn, D.E.P. Souto et al. introduced a self-made 3D matrix based on the PAMAM dendrimer with an ethylenediamine core for antigen immobilization in the competitive SPR immunoassay [83]. Due to the development of the surface available for the immobilization of the C1 protein (acting as immobilized antigen) by the introduction of the dendrimer, a significantly improved immobilization efficiency was achieved. According to the authors, the immunosensors in the 3D layers showed more than twice the sensitivity of the assay, compared to the classic immobilization on cysteamine SAM via glutaraldehyde chemistry.

**Figure 4:**
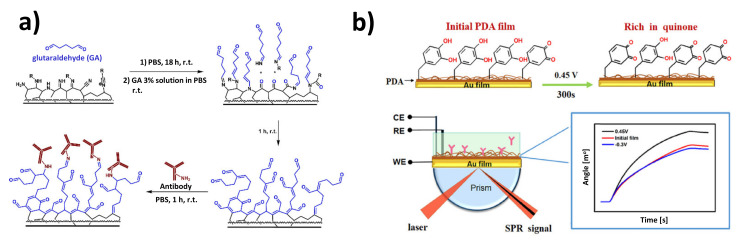
Examples of polymeric interphases employed in SPR immunosensors. (**a**) amine-terminated cyclopropylamine plasma polymer, (**b**) quinone-terminated polydopamine film. Adapted with permission from Makhneva et al. [84] (Copyright © (2018) Elsevier) and Chen et al. [89] (Copyright © (2018) Elsevier).

Another example of an amine-terminated polymer coating for the immobilization of antibodies has been described by Makhneva et al. The plasma polymerization of cyclopropylamine resulted in the coverage of the SPR slide surface with a polymer coating rich in nitrogen-containing functional groups. The surfaces were activated with glutaraldehyde, and after the subsequent immobilization of the antibodies, the obtained SPR immunosensor was employed to detect microbial cells. As shown in Figure 4a, the developed methodology for amine-bearing polymer coating can be successfully applied for oriented Ab immobilization via protein A or the amine/thiol-reactive linker, SMCC [84]. By changing the polymerization precursors to an acetylene-maleic anhydride mixture [85] and 1,2,4-trivinylcyclohexane (TVC)-tetrahydrofurfuryl methacrylate (THFMA) [86], the same group also synthesized carboxyl-rich copolymer films and used them as matrices for covalent antibody immobilization. It was confirmed that the binding capacity of such layers exceeds mercaptocarboxylic acid SAMs and is comparable to commercial CMD layers, and the obtained LODs for HSA detection were similar to MUA and 3D dextran-based sensors.

In another approach, a thin film of in situ polymerized dopamine was introduced as a passivating layer of an SPRi transducer (Figure 4b). The oxidative, UV irradiation-mediated polymerization of the deposited dopamine allowed a layer with catechol and quinone moieties to be obtained. Due to their reactivity in the Michael addition and formation of Shiff bases, this substrate can be used to effectively immobilize proteins through sulfhydryl and amino groups, without the need for prior pre-activation [87]. The authors showed the applicability of this substrate for the immobilization of the protein antigen-ochratoxin A (OTA) conjugate in the construction of a sensor to detect specific antibodies [88]. A similar layer, but obtained by electropolymerization, has also been applied for the SPR immunosensing of prostate-specific antigen [89].

### 2.4. Carboxymethyldextran Hydrogels and Planar CMD Surfaces

Carboxymethyldextran (CMD) 3D hydrogels and planar CMD surfaces are considered the gold standard within matrices for SPR immunosensing and thus deserve a separate discussion. Dextran coatings owe their commercial success to the ease of maintaining the form of a hydrophilic hydrogel, the facility of a controlled introduction of carboxyl groups into the structure and thus the regulation of the binding capacity, as well as the ability to control the polymer morphology. Thanks to this, it is possible to obtain universal hydrogel CMD coatings with different densities, degrees of branching, and surface charges. CMD-modified substrates are highly compatible with EDC/NHS surface chemistry. Thanks to the possibility of using a pH-adjustable protein preconcentration on the surface of previously activated sensors, it is possible to additionally increase the efficiency of covalent immobilization. In turn, the possibility of using zwitterionic CMD matrixes of a low charge density enables the efficient immobilization of negatively charged molecules. Another easily tunable parameter is the length of the dextran chains. For the detection of high-mass analytes, planar matrices are typically used. In the case of the detection of small molecules, to increase the receptors’ surface density (which determines the sensitivity of the assay), brush-like, 3D matrices with chain lengths of up to 700 nm can be employed. In such sensors, the hydrogel layer swells. An extensive spatial structure of the polymer matrix promotes the possibility of target association within a 3D brushed structure and thus enhances the RI changes responsible for the SPR angle shift as much as possible. Other examples of hydrogel materials that found applications as antifouling matrices of a high binding capacity include oligo(ethylene glycol) methacrylates, e.g., MeOEGMA, gelatin-based polymers, and linear polycarboxylates [90]. The use of CMD matrices, despite the obvious benefits of commercial availability and a high binding capacity, carry some risks. It is reported that CMD polymer substrates are characterized by a noticeable heterogeneity, resulting in the formation of regions of uneven affinities and inhomogeneity in terms of mass transport processes [91]. For this reason, the conscious selection of the substrate architecture in terms of the surface density and length of polymer chains is so important from the point of view of both characterizations of molecular interactions and SPR-based biosensing.

CMD substrates can be used as platforms for the immobilization of both antibodies and protein antigens, as well as for small molecule receptors used in indirect assay formats [92,93]. For example, He et al. employ the classic carboxymethyldextran (CM5) medium and EDC/NHS activation to immobilize the carrier protein conjugate (ovalbumin) with antigen 3-Nitrotyrosine (3-NT). The as-prepared receptor layer allowed for the SPR-based detection of this inflammatory biomarker in the competitive assay. The analytical parameters offered by SPR biosensors exceeded the parallelly developed ELISA [94]. The robustness of the covalent immobilization, together with the a capability of an efficient regeneration, results in the wide application of CMD polymer substrates for the detection of multiple analytes important from the point of view of medical diagnosis [95].

### 2.5. 2D Nanomaterials—SPR Interphases as Enhancers of the Plasmonic Signal

**Figure 5:**
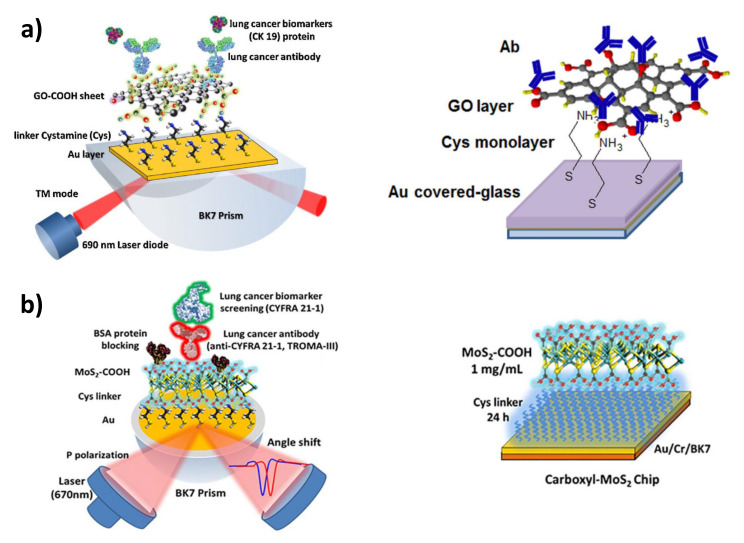
Carboxylated 2D nanomaterial layers as plasmonic enhancers in the construction of SPR immunosensors: (**a**) carboxylated graphene oxide, (**b**) carboxylated MoS2. Adapted with permission from Chiu et al. [98] (Copyright © (2018) Elsevier), Miyazaki et al. [102] (Copyright © (2018) Elsevier), and Chiu and Yang et al. [106] (under the terms and conditions of the Creative Commons CC BY License).

The unique optoelectronic properties of graphene have found numerous applications in modern bioanalytics, including the improvement of signal transduction in SPR biosensing. Ultra-thin layers of graphene offer numerous benefits for SPR sensors due to their high in-plane electron mobility and zero-band gap properties [96]. Covering the surface of the gold transducer with a thin layer of this material (e.g., by chemical vapor deposition) enables a significant increase of the SPR signal due to its capability to amplify refractive index changes. At the same time, the graphene surface plays a role in acting as a convenient platform for the immobilization of antibodies. The developed bifunctional linkers are based on pyrene derivatives implying a π−π interaction with aromatic rings of the graphene substrate. The use of the commercially available linkers, 1-pyrenebutyric acid or its NHS ester, enables a useful way to obtain amine-reactive surfaces for the rapid immobilization of antibodies [97]. Graphene can also be functionalized both chemically as well as by adsorption, thus opening a way to directly or passively immobilize through interactions of the hydrophobic protein domain [98,99]. Several methods for the oriented immobilization of antibodies/antigens on graphene, assisted by carrier proteins, such as A/G protein or avidin-biotin interaction, have been recently developed [64]. The introduction of water-dispersible graphene derivatives, such as GO and GO-COOH, simplifies the preparation of their layers on gold by enabling the deposition of the nanosheets directly from aqueous solutions [100]. To attach GO nanoflakes to the Au surface, cationic adhesive layers are commonly used, such as cysteamine, 3-mercapto-1-propane-sulfonate and cationic polymer PDDA [64,74,101,102,103]. The binding mechanism involves electrostatic interactions and reactions of GO epoxy groups with terminal amino groups on SAM-functionalized gold [64]. Examples of applications of 2D nanomaterial layers as plasmonic enhancers in the construction of SPR immunosensors are illustrated in Figure 5.

Apart from graphene and its derivatives, molybdenum disulfide (MoS_2_) has become another 2D material that significantly promotes plasmonic phenomena at the metal-dielectric interface. MoS_2_ is over two times more efficient than graphene in terms of optical absorption. This nanomaterial has been recently used in several SPR-based approaches [104]. SPR immunosensors were developed for the detection of biomarker proteins using both direct immobilization [105], coupling via terminal -COOH groups introduced through sulphur vacancies [106], and the decoration of nanomaterial deposited in the form of nanoflowers on the surface of the SPR transducer with gold nanoparticles [107].

2D Nanomaterials with a structure similar to graphene, which has attracted tremendous attention in recent years are transition metal carbides/carbonitrides (MXenes). Due to the high content of oxygen functional groups, MXenes are convenient substrates for the immobilization of biomolecules and, at the same time, make a significant contribution to SPR signal amplification [108]. The first applications of Ti_3_C_2_ MXene nanosheets in SPR immunosensors were described in 2019 and 2020 by Cui’s group. They developed sandwich-type SPR biosensors for the ultra-sensitive determination of carcinoembryonic antigen. The proposed, sophisticated mechanism of amplification and detection of plasmonic signals covered the decoration of MXenes by gold nanoparticles [109] or hollow nanoshells [110], followed by the protein A-assisted immobilization of *anti*-CEA antibody. The additional labelling step provided in sandwich assay format allowed the authors to determine CEA at the clinically relevant levels with a high sensitivity. Examples of the harnessing of selected 2D nanomaterials as substrates and signal enhancers in SPR immunosensors are depicted in Figure 5.

## 3. Affinity-Based, Oriented Ab Immobilization Strategies

As is well known, covalent immobilization carries an obvious risk of deteriorating the binding properties of immunoreceptors. What is more, chemical coupling through specific protein functional groups, such as -NH_2_, -COOH, or -SH, requires the use of high-purity antibodies/antigens and often results in the need for additional purification by the user. Therefore, methods involving chemical or biological affinity have become an interesting alternative to non-selective, chemical, or physical immobilization. The mild and targeted attachment helps to ensure the optimal spatial orientation of the recognition element, facilitates the control of their surface density, and gives greater freedom in the choice of reaction medium [111,112]. Over the years, many strategies of oriented antibody or recombinant antigen immobilization have been developed. Most of them have been implemented in the construction of SPR sensors from other methodologies. This was the case for the methods adapted from affinity chromatography for the purification of antibodies (proteins A, G, A/G) [113] and recombinant proteins (nitrilotriacetic acid—bivalent ion chelation via terminal His-tag). Other affinity-based methods, such as attachment via biotin tags or ssDNA anchor sequences, require the post-synthetic labelling of bioreceptors. Very good, critical discussions on various aspects of the design of immunosurfaces for plasmonic sensing have been presented in several reviews, including works provided by De Angelis [66], Mauriz [42], and Cheng [35]. The possibilities offered by affinity-based methodologies in the construction of SPR immunosensors for medical diagnostics, primarily in terms of universality, miniaturization, and multiplexing, will be discussed in the following sections.

### 3.1. Mediating Proteins: A, G, and A/G 

Proteins A and G are derived from bacteria and possess a strong binding activity for IgGs. That is why these biomolecules—commonly known as superantigens—offer the convenient, site-oriented immobilization of immunoreceptors [114]. Regardless of the specificity of Fab regions, it is possible to obtain highly bioactive layers with exposed binding sites. Bacterial protein A is a five-binding-domain protein isolated from *Staphylococcus aureus*. It captures the Fc portion of humans’ and domestic animals’ antibodies, leaving the Fab fragment accessible for the detection of the antigen [112]. Streptococcal protein G, which is similar in terms of functionality, contains repetitively arranged domains, where the COOH-terminal domains bind IgG, and the NH_2_ half residue was found to attach to human serum albumin. Thus, protein G has a broader spectrum of binding activity than protein A [115]. Nevertheless, native protein G is able to bind the Fab region of the antibody, but with an affinity 10 times lower than that towards the Fc region [116]. At first, these molecules were widely used in affinity chromatography for immunoglobulin purification. Therefore, their usage for the directed immobilization of antibodies was just a matter of time. As a non-specific binding between the surface of the metal and bare protein A is likely to appear, unmodified protein is not a preferable method for immobilization, and other chemical approaches typically need to be employed. The possibility of manipulating its affinity to antibodies and gold surfaces through genetic engineering, as well as its compatibility with various pre-immobilization strategies (e.g., carbodiimide coupling, thiolate, and dithiocarbamate self-assemblies), are behind the widespread use of this bacterial protein in the construction of plasmonic biosensors and commercial SPR substrates [66].

**Figure 6:**
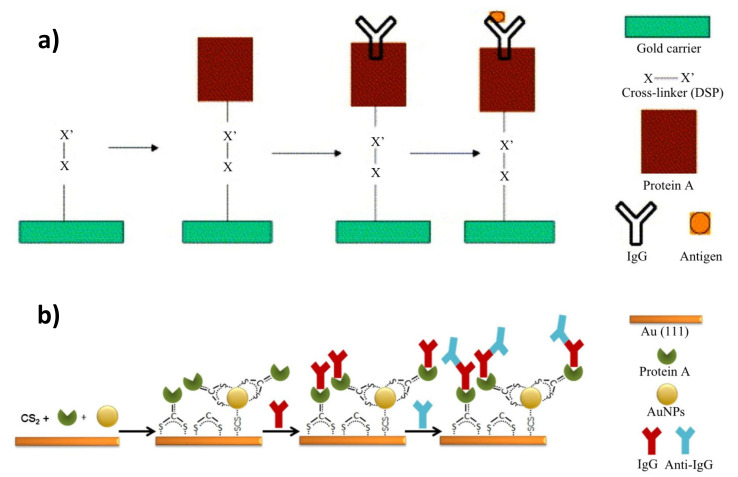
(**a**) Scheme of an SPR immunosensor construction based on the attachment of protein A to a gold surface through bifunctional DSP as a cross-linker, (**b**) functionalization of the terminal amino groups of protein A with carbon disulfide and direct immobilization of the carrier on gold by means of a dithiocarbamate assembly. Adapted with permission from Schmid et al. [120] (Copyright © (2006) Elsevier) and Paiva et al. [123] (Copyright © (2017) Elsevier).

In 2003, Lee et al. fabricated an immunosensor that determined bovine serum albumin in a probe. The device was based on a self-assembled monolayer of modified protein A, where the surface group of the compound was substituted with thiol functionality. The modified layer resulted in an increased antibody binding capacity and, with this, showed a better capture of the antigen [117]. Bakhmachuk et al. developed a biosensor that was dependent on the recombinant Staphylococcal protein A, with an added cysteine residue. Genetic engineering enabled the introduction of the attachment site into an expendable part of the protein, which increased the immobilization level of the molecule. The interaction between the thiol group and the surface of the gold sensor chip is much stronger and more reliable than physical adsorption [118]. Juan-Franco et al. also investigated the ability of modified protein A to form an appropriate layer for enhanced immunosensing. The staphylococcal immunoglobulin-binding domains were fused with a gold-binding peptide (GBP) forming PAG—the protein A gold-binding domain. This modification provided an easy and directed immobilization of the antibodies against human growth hormone, where Fab fragments remained freely exposed to the epitopes [119]. Schmid et al. used thiol-based homobifunctional cross-linker dithiobissuccinimide propionate (DSP) to covalently immobilize protein A on a gold surface. A sterically accessible, stable, and uniform antibody coating was achieved (Figure 6a) [120]. Sohn et al. also formed a layer of protein A on a chip modified with (3-aminopropyl)triethoxysilane (APTES), using a cross-linker (EDC-NHS) for the site-directed immobilization of the antibodies, and compared the results with non-activated protein A and a self-assembled monolayer [121]. The greatest sensitivity of the chip was obtained with protein A activated already with the cross-linker. The use of a cross-linker is relatively easier in comparison to engineered protein G or A, and it allows for antigen detection at very low concentrations. Streptococcal protein G was utilized by Hsu et al. to form a covalently attached layer for the immobilization of monoclonal antibodies. Three types of antibodies against pentamer C-reactive protein (CRP) and modified CRP were used for the subsequent detection of these molecules. No false signals were observed, and a sufficient sensitivity for the detection of this biomarker in biomedical samples was acquired [122].

In another approach, to increase the affinity of protein A to a gold surface without the need for a complex chemical modification or the use of recombinant protein, a simple, one-pot covalent immobilization via dithiocarbamate (DTC) chemistry was proposed [123,124]. In this strategy, the carrier protein was reacted with aqueous, alkaline carbon disulfide, which converts primary and secondary amino groups of protein A to dithiocarbamate. This simple functionalization allowed DTC-terminated molecules capable of bivalent interactions with gold to be obtained, involving the introduced sulphur atoms. The use of protein A functionalized the DTC chemisorption on flat transducers [124] and on the surface modified by introducing gold nanoparticles [123], which paved a way to the efficient immobilization of model antihuman IgG antibody and the use of an as-obtained platform in SPR-immunosensing (Figure 6b). This method has been also considered for the attachment of another intermediate protein—avidin—to a gold surface [66]. However, to date, it has not been used for the direct immobilization of protein receptors.

Bacterial mediator proteins and their bioconjugates are often applied in combination with other strategies aimed at improving the versatility or performance of SPR immunsurfaces [42]. Jung et al. developed a biolinker, which was a conjugate of protein G and DNA for the site-directed immobilization of antibodies. Single-stranded DNA oligonucleotides were attached to a gold surface, which enabled the hybridization of the complimentary oligonucleotide coupled with protein G. This form of immobilization resulted in a better oriented layer and greater ability to bind an antigen in comparison to the chip, where protein G was immobilized via chemical bonding [125]. More examples of spatially resolved protein attachment via DNA-directed immobilization is contained in chapter 3.4. Oh et al. fabricated a monolayer of protein G due to the chemical binding on a gold surface, which was modified with 11-mercaptoundecanoic acid (MUA). Antibody immobilization through protein G resulted in the construction of immunosensors for the detection of four kinds of the pathogens: *E. coli*, *Y. enterocolitica*, *S. typhimurium*, and *L. pneumophila* [126].

To further improve the anti-fouling properties of an antibody layer immobilized through thiolated protein A and backfilled with 3-mercapto-1-propanol, an additional, post-immobilization surface passivation with a cationic lipid membrane composed of ethylphosphocholine (EPC+) was also employed. The devised receptor layer combines the advantages of oriented antibody immobilization with an improved resistance to the adsorption of components of undiluted human plasma. The applicability of such an immunosensor for the detection of IgG antibody and cholera toxin in plasma samples was confirmed [127]. Biomimetic immunosensing interfaces based on lipid bilayers have also been implemented to functionalize SPR transducers. Almeida et al. described a layer made of common, amine-terminated phospholipids and cholesterol, which exhibits a high resistance to non-specific adsorption. The deposition of an appropriate composition of previously prepared lipid vesicles allowed for a rapid formation of bilayers, which are flexible but stable enough to be implemented in SPR measurement conditions. The additive of 5% decanethiol was responsible for the anchoring of the bilayer to the gold substrate, while exposed phosphate groups enabled the immobilization of protein through amine coupling, with the use of EDC/NHS chemistry [128].

### 3.2. Bacteriophages

Bacteriophages, also known as phages, are viruses that infect bacteria and the most abundant form of life on the planet [129]. They are extremely stable, and it is very easy to modify them, both genetically and chemically [130]. Due to these characteristics, they have become of considerable interest as probes for biosensing. Non-lytic and filamentous phages (those which do not lyse bacteria immediately after infection, are able to incorporate their genome into the host cell, and remain latent for a very long time) can be used as scaffolds for the binding of target moieties after chemical or physical functionalization. Both lytic and non-lytic genetically engineered phages were fabricated as an element of a biorecognition layer, and they can thus be successfully implemented in the construction of affinity biosensors [131].

Phage-derived biorecognition elements, such asphage-display peptides (the most recent) and elder, phage receptor-binding proteins or whole viral cells, are becoming more and more popular due their easy production, simple immobilization, rigidness, and ability to withstand very harsh conditions, such as high temperatures [132]. It is possible to insert the gene encoding the protein of interest into the phage’s genome, and then this particle is displayed as a fusion protein in the coating of the bacteriophage. Phage display is widely used for screening, but this technique is also suitable for biosensing as-obtained phage clones demonstrate peptides, proteins, and antibodies with the desired affinity for the target. It has been shown that phage display-identified peptides frequently bind to functional sites of the target proteins, rather than incidentally. Thus, non-specific interactions are rare [133]. Moreover, as there is a charge difference between the head of the bacteriophage (negative) and its tail fibers (positive) the site-directed binding using electrostatic forces is one of the most popular approaches of phage immobilization, after covalent coupling [134]. The general application of bacteriophages towards biosensors may be grouped into three categories: (a) specific peptides displayed on the surface of non-lytic phages’ coats due to genetic engineering for target analyte detection; (b) lytic bacteriophages that disrupt the host, causing a release of bacterial cell markers and, afterwards, their detection (phage acts like an antibody); and (c) phages as scaffolding material for the immobilization of functional molecules [135].

Nanduri et al. developed a real-time biosensor, which was based on the layer of phage 1G40 immobilized on the gold surface of an SPR chip via physical adsorption. The detection of beta-galactosidase was possible due to the specificity of filamentous bacteriophage to this model antigen [136]. In the same year, they examined an SPR immunosensor for the identification of L. monocytogenes. Bacteriophage Lm P4:A8, which expressed a single-chain variable fragment antibody against transmembrane protein exposed on the bacterial surface, was immobilized on the chip due to physical adsorption. Low detection limits were achieved, and the whole process was effective and simple, as it did not require additional reagents or complex chemistry [137]. Karoonuthaisiri et al. used filamentous phage M13, which expressed 12-mer peptides for the detection of *Salomonella* [138]. Naidoo et al. studied a similar approach, and they used immobilized phages T4 and P22 for bacterial detection. Nevertheless, the adsorption to the surface was highly heterogeneous due to the phage clustering at higher surface densities, which ultimately limited the ability to capture the target [139].

Unluckily, phage display is a laborious and time-consuming technique requiring skilled researchers and additional equipment. Therefore, phage-based layers used in SPR immunosensors are not common and well-studied yet. However, their simplicity, robustness, rigidness, and stability make them a valuable and promising approach. However, there is a phage for each bacteria. Thus, bacteriophages are a cheap alternative to antibodies. Nonetheless, an attractive aspect is the possibility of using phages in the current development of immunosensors. Phage displays are gaining popularity as tools for the selection of synthetic antibodies or for screening antibody-biomarker interactions for both therapeutic applications and immunosensing [140,141].

### 3.3. Biotin—(Strept)Avidin Affinity

Biotin (Bt), also known as vitamin H, is a tiny water soluble molecule produced by many prokaryotic organisms and plants [142]. Biotin/avidin or biotin/streptavidin interlinkage is a non-covalent method of substrate immobilization to the chip surface with a high affinity. Biotinylation—attachment of the biotin to a protein, such as an antibody—can be done both in vivo and in vitro [143]. Biotinylation in vivo is accomplished at the genetic level by fusing the gene encoding the protein of interest to the gene-coding biotin, i.e., the desired fusion tag. This phenomenon uses microbial vectors and tools of genetic engineering. Tagging in vitro can be obtained through the usage of chemical reagents or enzymes, such as biotin ligase from *E. coli* [144]. Streptavidin (Sa) and avidin (Av) are thermostable homotetramers that bind molecules of biotin with K_d_ ~10^−14^ M (streptavidin) and K_d_ ~10^−15^ M (avidin). The first one is derived from *Streptomyces avidinii*. It is resistant to a high pH and is intransigent to degradation when exposed to enzymes and denaturing agents. Avidin is extracted from the oviparous vertebrates’ eggs. The non-covalent interaction between biotin and these proteins is of great strength and specificity. It is approximately 10^3^ to 10^6^ times greater than antigen–antibody binding [145,146]. As avidin possess additional carbohydrate moieties—three N-acetyl glucosamine residues and four mannose in each unit—its pI is higher, resulting in a positive charge of the physiological pH, which causes a non-specific binding to the surfaces and molecules of the negative electrical charge, even though its interaction with biotin is the strongest biological binding ever described [147,148]. The immobilization of the protein of interest on the chip surface through the biotin/(strept)avidin interaction is simpler in comparison to covalent coupling [149], and (strept)avidin-coated sensor chips are commercially available (Biacore, Reichert Technologies, XanTec, Sofchip).

**Figure 7:**
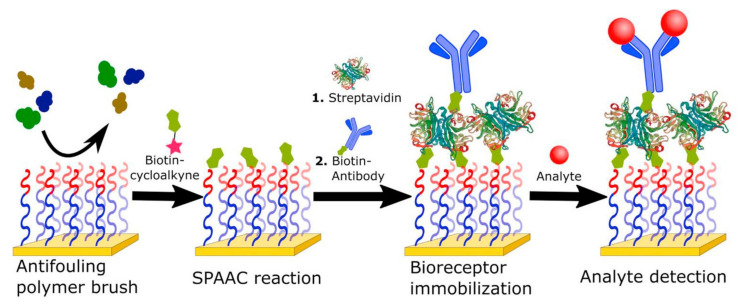
Schematic of a hybrid, multilayer SPR substrate composed of an antifouling polymer brush and biotin-captured streptavidin for the immobilization of biotinylated antibody. Adapted with permission from Parrillo et al. [90] (Copyright © (2017) Elsevier).

Dutra et al. used a carboxymethyldextran-modified gold chip covered with covalently coupled streptavidin forming a monolayer for the attachment of biotin-tagged anti-troponin T monoclonal antibodies. An immobilized layer together with an SPR apparatus served as an immunosensor for human cardiac troponin T. This method enables identification in real-time, with a detection limit around 0.01 ng/mL, which is comparable to ELISA methods [150]. There are many other approaches where biotin/streptavidin interactions were used together with SPR to form an immunosensor for the detection of moieties of biological origin or for studying the interplays between them, e.g., markers [151], heparin [152], hormones [153], and viruses [154,155]. An example of a hybrid architecture combining the use of oligo(ethylene glycol) methyl ether methacrylate backfilling and affinity-based immobilization via avidin-biotin interaction is the SPR biosensor described by Parrillo et al. The employment of a thick polymer brush reduced non-specific adsorption, thus enabling analysis of blood plasma. At the same time, the biotin tag, which has been easily introduced by alkyne-azide cycloaddition, opened the way to the fast and effective anchoring of labelled immunoreceptors (Figure 7) [90].

Beyond receptor immobilization, biotin/streptavidin interactions are also used to increase SPR signals [156,157,158,159]. Usually, the use of a dextran matrix results in its enhancement, but it does not affect the improvement of the sensitivity. Thus, analytes of a low molecular mass become undetectable. Pei et al. checked that the biotinylated protein-streptavidin complex is massive enough to amplify the response signal, inducing a boost of the detection sensitivity. In their studies, human immunoglobulins were detected at a level of 5 ng/mL [158].

In 2020, Sun et al. proposed another strategy, using biotin/streptavidin interlinkage to enhance the SPR signal. They applied “one-pot”-prepared bioconjugates of streptavidin-tagged antibodies and biotin-labeled phenylalanine nanoparticles. They prepared biotinylated phenylalanine monomers, which, in mild conditions, were able to self-assemble into nanoparticles. Subsequently, these nanoparticles were used as transporters of the complexes of antibodies tagged with streptavidin due to the strong biotin/SA interaction. Because of the high molecular weight of the conjugates, the SPR signal was amplified. Prostate-specific antigens were used to determine goal concentrations as low as 1 pg/mL [160].

Due to the high binding affinity of biotin and streptavidin, the process of their dissociation is extremely difficult. Consequently, reusing streptavidin-modified surfaces is practically unachievable. Li et al. described a new approach that enables the reuse of a streptavidin-coated SPR biosensor chip. They tagged an antibody against mature bovine prion protein with a nano-tag and streptavidin-binding protein (SBP) [161]. A nano-tag is a dozen amino acids of long protein that possess a constant dissociation below 17 nM while binding to streptavidin [162]. SBP is a 38 amino acid tag of K_d_ ~2.5 nM, which can be eluted natively with biotin, enabling conditions for the purification of the proteins [163]. As both proteins have a weaker capacity of the streptavidin attachment, but they remain highly specific, it is possible to perform repeatable regenerations with a diluted NaOH solution, without losing the activity of the chip [161]. Another approach utilizes captavidin—a derivative of avidin that possess nitrated tyrosine in the site-binding biotin. While the specificity of the interaction remains the same, this modification enables the easy dissociation of the complex at pH 10.0. Garcia-Aljaro et al. showed that captavidin can be immobilized on the surface of the SPR sensor chip and then effectively take up to nine serial capturing and regeneration steps [148]. Regenerable (strept)avidin has become of great interest, and nowadays, researchers are discovering new mutants for the best combination of the properties, such as a high binding capacity, low level of nonspecific adsorption, and good stability [164]. 

The attachment of the biotin molecule to antibodies typically requires a separate, in vitro reaction, with the use of previously purified immunoglobulin. Despite this complication, the unmatched robustness of this interaction, as well as the multivalence of the avidin molecule (containing four binding sites), opens up many possibilities of using both simple (Av-modified surface + biotinylated receptor) and sandwich formats (Bt-modified surface + regenerable avidin + biotinylated receptor—see Figure 7). As shown above, a particularly interesting trend increasing the suitability of the platform for future SPR-based diagnostic applications is the use of an Av-Bt interaction, in combination with other solutions, with the aim of improving the performance parameters of immunosensors, including site-oriented immobilization, fouling-resistant layers, and SPR sensitivity enhancement.

### 3.4. DNA- and Antibody Directed Immobilization

Along with the development of automation and the progressive miniaturization of plasmonic sensors, new aspects have been playing an increasingly important role in recent years. The possibility of the fabrication of universal and fully regenerable receptor layers seems to be at the forefront. This idea allows the difficulties related to the sensitivity of the immunoreceptor layer to—often harsh—conditions required to destroy the antibody-antigen interaction to be overcome. In such a case, an attractive alternative to regeneration is the removal of the entire receptor layer and its reconstruction with the use of rapid and controllable methods of molecular self-assembly, such as “click biology”. Beyond the already mentioned regenerable avidin, this strategy can be also accomplished by means of DNA- and antibody-directed immobilization [165].

Immobilization of the proteins through DNA was first described in the early 1990s. Due to the large-scale production and physicochemical stability of this molecule, and therefore its easy storage and widespread usage, DNA has become of interest in relation to site-directed immobilization on solid supports. This nucleic acid does not affect the biological activity of the protein, and the process of the conjugation requires mild conditions. Moreover, DNA is a great anchor, because it behaves like a rigid elastic rod in hydrated ionic liquids [165,166]. Oligonucleotides carrying thiol groups can be simply attached to the gold surface of an SPR chip, and then Watson-Crick base pairing enables the hybridization of a complimentary oligonucleotide, resulting in the immobilization of the DNA-tagged protein of interest. The main advantages of DNA-directed immobilization (DDI) are its specificity, stability, effortlessness, versatility, high surface coating density, and the possibility of regeneration via a simple denaturation protocol [165].

In 2004, Ladd et al. constructed an SPR biosensor with a functionalized surface for multichannel detection. The self-assembled monolayer of oligoethylene glycol and biotinylated alkanethiol on the gold chip enabled the coupling of single-stranded DNA through a streptavidin bridge. The DNA-tagged antibody against human chorionic gonadotropin (hCG) was immobilized on the surface by a hybridization process [167]. Four years later, they used a similar approach for the simultaneous detection of the DNA and proteins in the probe. Instead of additionally using a biotin-streptavidin bridge, thiolated oligos were used for micro-spotting. The biosensor consisted of eighteen spots for the identification of four different DNA sequences and for two proteins: human chorionic gonadotropin (hCG) and follicle stimulating hormone (FSH). For the detection of hormones, conjugates of an appropriate antibody and complimentary DNA were used [168]. Bombera et al. designed and constructed a DNA-DNA-antibody SPR biochip for real-time cell sorting. Using the same device, it was possible to investigate both molecular and cellular interactions. Anti-CD19 and anti-CD90 IgGs were chosen for capturing B and T lymphocytes, respectively. The utilization of restriction endonucleases enabled the enzymatic cleavage of DNA-protein conjugates, resulting in the monitored release of the cells [169]. Leroy et al. examined an analogous approach to DDI for cell sorting. Additionally, a sensor chip was assembled in a microfluidic device, which was integrated with SPRi. Targeted release was possible due to the laser-induced heating [170]. Piliarik et al. has described the SPR immunosensor exploiting site-selective DNA-directed immobilization of antibodies against human chorionic gonadotropin (hCG) and activated leukocyte cell adhesion molecule (ALCAM), as biomarkers abundant in blood plasma [171]. DNA conjugates of low-molecular-weight antigens were also employed in the construction of SPR biosensors in a competitive format, as in the case of the immunosensor described by Tort et al., where a steroid–oligonucleotide conjugates line was used as recognition elements [172].

**Figure 8:**
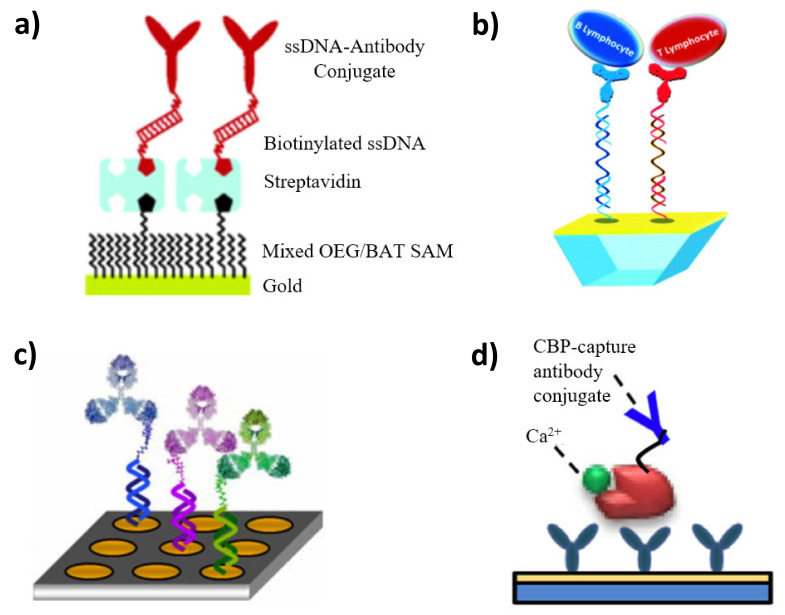
Various concepts of DNA-directed immobilization in the construction of SPR immunosensors: (**a**) immunosensor based on DNA anchoring via Av-Bt affinity, (**b**) direct SPRi assay for the detection of lymphocytes, employing DNA hybridization in sandwich-like format, (**c**) competitive SPRi immunoassay with the use of DNA-steroid conjugates, (**d**) immunosensor using Ca^2+^-dependent antibody-directed immobilization. Adapted with permission from Ladd et al. [167] (Copyright © (2004) American Chemical Society), Leroy et al. [170] (Copyright © (2004) Royal Society of Chemistry), Tort et al. [172] (Copyright © (2012) American Chemical Society), and Kim et al. [173] (Copyright © (2017) Elsevier).

A similar system based on antibody-directed immobilization (ADI), which offers the rapid regeneration of the receptor layer dependent on the reversible antibody–antigen interaction was described by Kim et al. The developed immunoreceptor layer for the real-time monitoring of biomarkers involves the Ca^2+^ ion-dependent binding of the calcium-binding protein (CBP) conjugate and a monoclonal antibody specific to creatine kinase-MB. The Ca^2+^-switchable capturing of CBP by an auxiliary, surface-tethered antibody enabled the facile and reversible control of the engagement and disengagement of the captured antibody conjugate on the SPR chip surface. This approach does not require the use of harsh conditions during the regeneration step and thus preserves the long-term viability of the receptor layer. The authors provide an universal system based on the exchangeable capture antibody for semi-continuous immunosensing [173]. Another example of antibody-directed immobilization is the use of *anti*-his-tag antibodies. Such versatile immunosurfaces show the ability to bind a wide range of recombinant proteins and antibodies, while offering the simplicity of their replacement by means of conventional regeneration methods known from antibody–antigen interactions. This solution is currently used in the SPR screening of new variants of polyhistidine-tagged scFvs [174]. However, in the near future, it will also be possible to implement it for immunosensing. The selected formats and applications of DDI and ADI in plasmonic immunosensing are shown in Figure 8. 

The prospect of creating multiplex immunoassays is of exceptional value, as the conventional fabrication of arrays for the detection of dissimilar proteins requires different chemistries of the surface. The greatest challenge of DDI that has to be faced is to find the method of oligo-protein conjugation that possess a high efficiency and, subsequently, has a simple and rapid purification. Additionally, the coupling of multiple DNA strands to a single protein is unlikely to be avoided. Thus, the technique for obtaining the highest yields from mono-conjugated products is costly. In recent years, “click” reaction copper-free alkyneeazide cycloaddition using cyclooctyne (DBCO) reagents turned out to be a promising alternative to the classic carbodiimide and maleimide coupling in the biofunctionalization of protein receptors using various components and tags (including ssDNA) [90,175,176]. Together with the dedicated systems of chromatographic purification, this enabled the popularization of DNA-protein conjugates in the construction of immunoarrays and their use in commercial systems for studying intermolecular interactions [177,178].

### 3.5. Bivalent Ion-Mediated Chelation of His-Tagged Receptors

Affinity tags have multiple advantages, such as having mild conditions for capturing, the capacity for site-directed immobilization, low price (usually), and the possibility of regeneration the chip. At the same time, they require the use of genetic engineering techniques. Apart from the use of substrates functionalized with super-antigenic proteins (proteins A, G, and A/G), another approach adapted from the methods of protein purification is immobilization with the use of a polyhistidine tag through bivalent ion-nitrilotriacetic acid (NTA) complexes [179]. This pathway is particularly convenient in the case of designing immunosensors in a competitive format, where recombinant proteins or synthetic oligopeptides are involved in the receptor layer. Importantly, when the receptor/analyte-binding mechanism is well known, it is also possible to design and deliberately insert a his-tag into such a region of the receptor, which will facilitate the process of oriented immobilization, without negatively affecting the binding sites [180].

Kimple et al. studied the protein–protein interactions in heterotrimeric G-protein biology using a His6 fusion tag for direct nonrandom immobilization [181]. Fischer et al. compared the attachment of a substrate-binding protein (SiaP) to the NTA surface of the sensor chip via C-terminal hexa-histidine (His), N-terminal deca-His, and N-terminal double-His tags. SiaP derived from *H. influenzae* possess a high affinity to N-acetylneuraminic acid, which is a metabolic marker during the progression of coronary artery disease [182]. The hexa-His tagged protein could not be stably anchored even at low flowrates and a low concentration of the protein, and the immobilization of highly concentrated deca-His tagged SiaP resulted in a baseline drift. The double-His tagged approach was preferred, as it provided a reliable baseline and prompt regeneration of the chip [183]. Chu et al. tagged the protein, CXCR5 (chemokine receptor), with 6xHis and an HPC4 tandem tag for the facilitation of both the purification of the protein and its immobilization on the chip. HPC4 is a 12 amino acid sequence, which encodes some of the residues of the protein C heavy chain. A monoclonal antibody kinetics assay was performed, and this peptide-based binding assay was further applied for the capture of virus-like particles [184].

**Figure 9:**
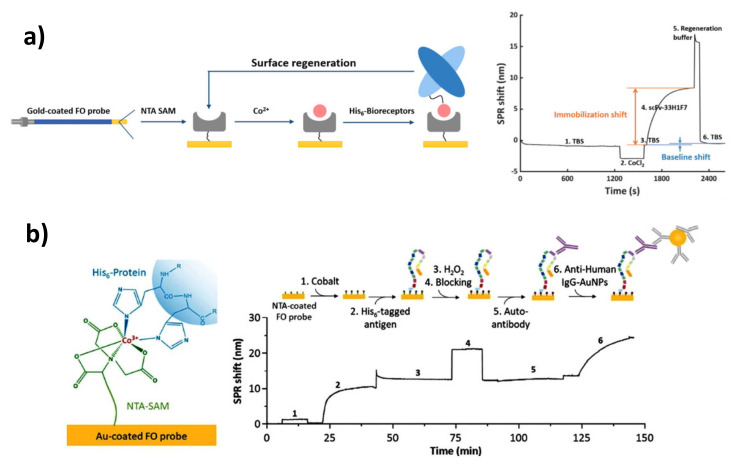
The application of cobalt ion-nitrilotriacetic acid complexes in SPR substrates for the immobilization of recombinant proteins. (**a**) Easily regenerable immobilization with a Co^2+^ ion, (**b**) robust chelation with a Co^3+^ ion. Adapted with permission from Qu et al. [187] (under the terms and conditions of the Creative Commons CC BY License) and Horta et al. [186] (Copyright © (2020) American Chemical Society).

Due to the non-covalent and reversible mechanism of Ni^2+^-dependent complexation (high K_D_ values in physiological media), the disadvantage of this approach is the gradual dissociation of the receptor from the surface. An important improvement in the Ni^2+^-NTA-based immobilization of recombinant proteins was proposed by Wang et al. An additional step of the covalent affixation of bioreceptors with carboxymethyldextran-NTA residues via EDC/NHS coupling prevented the gradual degradation of the layer, while maintaining the homogeneous receptor orientation, which is determined by the previous chelation of His-tag-terminated sites. Thanks to this, it was possible to combine the advantages of covalent immobilization with the possibility of using a pre-concentration of tagged ligands on the surface and maintaining control over their orientation. This enables the construction of a competitive SPR biosensor for studying the interaction of non-small-cell lung cancer biomarker t-DARRP with a specific antibody [185]. To improve the stability of the receptor immobilization using the His-tag—in particular, the resistance to desorption in plasma samples—the Lammertyn and Vanhoorelbecke group proposed the chelation of the protein antigen on the fiber optic SPR sensor surface using Co^3+^, instead of Ni^2+^. The superiority of the Co^3+^-dependent His-tagged receptor chelation, relative to Ni^2+^, in terms of a better stability and sensitivity, was confirmed for the detection of autoantibodies, as a marker of autoimmunological disease [186]. In turn, the reversible, Co^2+^-dependent chelation of recombinant, His_6_-tagged single-chain variable fragments of *anti*-SARS receptor-binding domain (RBD) antibodies were used in the construction of a fiber-optic SPR biosensor. The authors confirmed the full reusability of the sensor probes on NTA media using various types of His-tagged bioreceptors, including recombinant antibody fragments and antigens [187]. Both Co-dependent mechanisms of the attachment of the His-tagged proteins are depicted in Figure 9.

### 3.6. Other Affinity-Based Methods for the Attachment of Recombinant Receptors

Recent trends in immunoreceptor engineering cover the development of receptors characterized by well-defined kinetic parameters [188,189]. The wide capabilities of the SPR and SPRi platforms would be fully exhibited in the case of their adaptation to biosensors operating in a continuous or *semi*-continuous mode. The monitoring of the concentrations of clinically relevant analytes, without the need for periodic receptor layer regeneration, requires the use of receptors characterized by fast association and dissociation kinetics (k_a_ and k_d_) and thus a relatively low affinity (high equilibrium constant K_D_, expressed as k_d_/k_a_). Classic methods of the selection and production of antibodies do not offer the possibility of the isolation of such receptors. However, the progress in the development and isolation of recombinant antibodies and their fragments open up completely new possibilities for the construction of SPR immunosensors capable of working in the real-time and continuous modes. In the case of the availability of sufficiently high-quality receptor-transducer interphases, *semi*-continuous immunosensing can be successfully introduced into routine medical and companion diagnostics [190]. It is worth emphasizing that for applications in *semi*-continuous plasmonic biosensors, fast and spatially resolved immobilization methodologies are desirable. They should enable the quick patterning of immunoreceptor arrays, as well as refreshing them through a simple exchange of the receptor layer. For this reason, site-oriented immobilization, which is based on affinity interactions (in particular, “chick chemistry” and “click biology”), seem to be ideally suited both to the needs of modern and flexible SPR and SPRi platforms for rapid screening, as well as the monitoring of clinically relevant analytes [191].

To increase the intrinsic affinity of antibody fragments to a bare gold surface, it is possible to fuse scFs fragments with gold-binding polypeptides by genetic engineering. Such a construct shows a rapid and oriented immobilization ability on the chip surface through an Au-S bond formation. Zhou et al. described recombinant hybrid fragments of anti-CFP-10 antibodies with histidine_6_-tagged gold-binding protein (6His-GBP) in an SPR immunosensor for the detection of *Mycobacterium tuberculosis*. Owing to the controlled orientation of the bioreceptor and the proximity of the Au surface, as well as the use of gold-magnetic nanoparticles, a significant amplification of the signal was noticed [192]. Ha et al. developed an SPR-based immunoarray, where antibodies (human IgGs) were immobilized on the glutathione-modified gold chip surface, which was covered with immunoglobulin-binding domains tagged with glutathione S-transferase (GST). The potential of GST-tagged proteins, as anchors for the site-directed immobilization of antibodies, was compared with the conventional covalent coupling method. It turned out that the SPR response of immobilized IgGs, as well as the capability of binding with the antigen, was greatly enhanced in the case of the first method [193]. Binding via a his-tag-NTA is less specific than via a GST-tag, but it is cheaper and broadly available [194]. While immobilization via tags is not a newly discovered approach in protein anchoring, its fusion with antibodies, such as nano- or affibody, or the use of brand-new affinity tags is a cutting edge. In 2020, Boonen et al. developed an SPR immunoassay for the investigation of interactions between chemokine receptor CXCR4 and a nanobody fused with an Fc fragment. This chemokine receptor is highly expressed in numerous types of tumor cells. These cells hijack its pathway, which enables the metastasis of the tumor in distinct sites of the organs. CXCR4 was tagged with a C-terminal histidine_10_ and successfully immobilized on a nitrilotriacetic acid sensor chip, which enabled the detection of nanobodies in the sample. The immobilized particles were stable and active for at least 12 h, the acquired kinetic data were reliable, and the surface was susceptible to removal during regeneration [194]. In 2017, Hussack et al. developed an antibody screening assay through SPR analyses. They used a new camelid-derived affinity tag, called ABTAG, which captures bovine serum albumin with a picomolar binding affinity. The immunization of llamas with carcinoembryonic antigen-related cell adhesion molecule 6 (CEACAM6) resulted in the production of heavy chain antibodies and then their use as a template for phage-displayed single-domain antibodies (sdAbs) against this protein. After panning (affinity selection technique in phage display), the expressed sdAbs were fused with ABTAG. Anti-CEACAM6-ABTAG conjugates were bound to the surface covered with BSA. The binding of the antigen to the complex enabled the determination of kinetic and affinity constants, as well as the read-out of the fusion protein expression level [195]. Examples of other promising receptors for applications in modern SPR immunosensing cover, e.g., recombinant antibodies and antibody fragments (polyhistidine-tagged scFvs [174], Fc-tagged scFvs [196], camelidae IgG-like molecules [197], nanobody-Fc (Nb-Fc) ligands [194], Fc-tagged chimeric proteins [198]), and mediating proteins (affinity-engineered staphylococcal Protein A and protein A fused with a gold-binding peptide [199]).

## 4. Conclusions

SPR and other plasmonic immunosensors in medical diagnostics are typically compared to other immunodetection methods. Biosensors with an SPR readout, as flagship representatives of label-free techniques, offer advantages in terms of detection time and simplicity in relation to methods employing enzyme immunolabeling, e.g., ELISA assays. Despite decades of SPR use in the detection of clinically relevant bioanalytes in real samples, the development of functional transducer-receptor interphases is still one of the main directions in the development of plasmonic immunosensors. Meanwhile, antibodies, as elements capable of the specific recognition of bioanalytes in complex samples, are still the gold standard. 

The main research topics outlined in this review are focused on the development of high-quality immunoreceptor layers. They should guarantee an appropriate efficiency in the immobilization of protein receptors, optimal spatial orientation, good sensitivity to changes in the local refractive index, and minimization of the effects associated with the non-specific adsorption from samples like body fluids. Since both the design of intermediate layers and the method of immobilizing biological receptors play an important role in achieving the abovementioned objectives, a thorough description of the recent and most exploited approaches in the field has been provided. This paper introduces and classifies approaches to the design of immunosensing platforms with the use of both random and site-oriented immobilization strategies. Additionally, hybrid methodologies—although difficult to clearly classify—were presented in this review. They usually combine the advantages of 3D substrates with site-oriented immobilization and the introduction of agents, ensuring ultra-low nonspecific binding capacity. Such versatile platforms turned out to be the most promising from the point of view of their feasibility in the field of medical diagnostics.

The continuous development of immunoreceptors is aimed at improving their affinity and compatibility with typical transducers. Antibodies characterized by ultra-fast kinetics of interaction with a target molecule, as well as the implementation of recombinant proteins as recognition elements, also opens up new possibilities for SPR immunosensors. The current trends, focused on the miniaturization and multiplexing of SPR platforms, as well as on the possibility of working in a (semi)continuous mode, pose new challenges in terms of the robustness and versatility of the design of sensor substrates. This results in the development of novel strategies, such as the fabrication of easily designable and regenerable layers via affinity-directed immobilization or the immobilization of recombinant, tagged receptors. Therefore, in the coming years, a great deal of scientific effort will be focused on the improvement of current and the design of new types of plasmon-based immunosensors. The constant pursuit of adapting SPR interphases to the possibilities offered by new receptors, like recombinant proteins and antibody fragments, aims to meet the requirements of modern medical diagnostics.

## Data Availability

Not applicable.

## Abbreviations

Ab	antibody
ADI	antibody-directed immobilization
ALCAM	activated leukocyte cell adhesion molecule
APTES	(3-aminopropyl)triethoxysilane
Av	avidin
Bt	biotin
CBP	calcium-binding protein
CEA	carcinoembryonic antigen
CEACAM6	carcinoembryonic antigen-related cell adhesion molecule 6
CFP	culture filtrate protein
CMD/CM5	carboxymethyldextran
DDI	DNA-directed immobilization
DSP	disuccinimido dithiobispropionate
DTC	dithiocarbamate
EDC	N-(3-dimethylaminopropyl)-N′-ethylcarbodiimide hydrochloride
EOT	extraordinary optical transmission
EPC	ethylphosphocholine
Fab	antigen-binding fragments
Fc	fragment crystallizable
FSH	follicle-stimulating hormone
GBP	gold-binding peptide
GO	graphene oxide
GST	glutathione S-transferase
HA	hyaluronic acid
HSA	human serum albumin
hCG	human chorionic gonadotropin
HEGD	hexa(ethylene glycol) dithiol
His	histidine
LSPR	localized surface resonance
MBA	mercaptophenylboronic acid
MeOEGMA	poly[oligo(ethylene glycol) methyl ether] methacrylate
MHDA	mercaptohexadecanoic acid
MPA	mercaptopropionic acid
MUA	mercaptoundecanoic acid
Nb	nanobody
NHS	N-hydroxysuccinimide
NT	nitrotyrosine
NTA	nitrilotriacetic acid
OTA	ochratoxin A
PAG	protein A gold-binding domain
PAMAM	poly(amidoamine)
PDDA	poly(diallyldimethylammonium chloride)
pHEMA	poly(2-hydroxyethyl methacrylate)
PNIPAAM	poly(N-isopropylacrylamide)
RBD	receptor-binding domain
RI	refractive index
Sa	streptavidin
SAM	self-assembled monolayer
SBP	streptavidin-binding protein
scFv	single-chain fragment variable
sdAbs	single-domain antibodies
SiaP	sialic acid-binding periplasmic protein
SMCC	succinimidyl 4-(N-maleimidomethyl)cyclohexane-1-carboxylate
THFMA	tetrahydrofurfuryl methacrylate
TVC	1,2,4-trivinylcyclohexane
